# Differential Expression of Prognostic Proteomic Markers in Primary Tumour, Venous Tumour Thrombus and Metastatic Renal Cell Cancer Tissue and Correlation with Patient Outcome

**DOI:** 10.1371/journal.pone.0060483

**Published:** 2013-04-05

**Authors:** Alexander Laird, Fiach C. O’Mahony, Jyoti Nanda, Antony C. P. Riddick, Marie O’Donnell, David J. Harrison, Grant D. Stewart

**Affiliations:** 1 MRC Human Genetics Unit, University of Edinburgh, Edinburgh, United Kingdom; 2 Edinburgh Urological Cancer Group, University of Edinburgh, Edinburgh, United Kingdom; 3 Department of Pathology, Western General Hospital, Edinburgh, United Kingdom; 4 School of Medicine, University of St Andrews, St Andrews, United Kingdom; Baylor College of Medicine, United States of America

## Abstract

Renal cell carcinoma (RCC) is the most deadly of urological malignancies. Metastatic disease affects one third of patients at diagnosis with a further third developing metastatic disease after extirpative surgery. Heterogeneity in the clinical course ensures predicting metastasis is notoriously difficult, despite the routine use of prognostic clinico-pathological parameters in risk stratification. With greater understanding of pathways involved in disease pathogenesis, a number of biomarkers have been shown to have prognostic significance, including Ki67, p53, vascular endothelial growth factor receptor 1 (VEGFR1) and ligand D (VEGFD), SNAIL and SLUG. Previous pathway analysis has been from study of the primary tumour, with little attention to the metastatic tumours which are the focus of targeted molecular therapies. As such, in this study a tissue microarray from 177 patients with primary renal tumour, renal vein tumour thrombus and/or RCC metastasis has been created and used with Automated Quantitative Analysis (AQUA) of immunofluorescence to study the prognostic significance of these markers in locally advanced and metastatic disease. Furthermore, this has allowed assessment of differential protein expression between the primary tumours, renal vein tumour thrombi and metastases. The results demonstrate that clinico-pathological parameters remain the most significant predictors of cancer specific survival; however, high VEGFR1 or VEGFD can predict poor cancer specific survival on univariate analysis for locally advanced and metastatic disease. There was significantly greater expression of Ki67, p53, VEGFR1, SLUG and SNAIL in the metastases compared with the primary tumours and renal vein tumour thrombi. With the exception of p53, these differences in protein expression have not been shown previously in RCC. This confirms the importance of proliferation, angiogenesis and epithelial to mesenchymal transition in the pathogenesis and metastasis of RCC. Importantly, this work highlights the need for further pathway analysis of metastatic tumours for overcoming drug resistance and developing new therapies.

## Introduction

Renal cell cancer (RCC) accounts for 2.5% of all adult cancers, but it is the most lethal of all urological malignancies. One third of RCC patients present with metastatic disease (mRCC). While extirpative surgery is intended to be curative in those with localised disease, a further 30–40% eventually develops distant metastases [1], [2], [3]. At present, we are unable to accurately predict those patients who will relapse, due to the highly variable natural history of the disease.

Following surgery, a number of prognostic nomograms have been proposed for predicting disease recurrence. These typically include TNM staging and Fuhrman grade as well as performance status and serum blood markers (haemoglobin, calcium, lactate dehydrogenase, platelets, neutrophils and C-reactive protein) to a greater or lesser extent [4], [5], [6], [7]. These clinico-pathological variables are widely used in clinical practice for stratifying risk of recurrence and planning follow-up. Despite this, none of these models are 100% accurate. In an attempt to improve patient’s risk stratification, and better predict disease recurrence and associated mortality, many have investigated the role of prognostic biomarkers [8], [9]. The EMT markers, SLUG and SNAIL, have been reported to have predictive and prognostic importance in RCC [10], [11]. However, most notably, Klatte *et al* conducted an elegant study of key proteins in RCC pathogenesis using the TMA technique to show Ki67, p53, vascular endothelial growth factor receptor 1 (VEGFR1) and vascular endothelial growth factor ligand D (VEGFD) were associated with disease free survival (DFS) in localised RCC [12]. This panel of markers combined, was able to better predict DFS compared to standard variables. A prognostic nomogram was constructed combining these markers with clinico-pathological variables for the first time showing an improved predictive accuracy. Despite this, the routine use of these markers in the clinic has not been adopted.

A better understanding of the molecular basis of RCC has not only advanced biomarker discovery, but also advanced the treatment of metastatic RCC (mRCC) with the development of targeted molecular therapies. The most important discovery has been the identification of mutations in the von Hippel-Lindau (VHL) gene in hereditary clear cell RCC (ccRCC) and up to two-thirds of sporadic cases [13]. This leads to activation of the hypoxia pathway and the subsequent up-regulation of hypoxia associated molecules i.e. vascular endothelial growth factors (VEGF) and platelet derived growth factors. These discoveries resulted in the clinical use of VEGF monoclonal antibody (bevacizumab) and multi-targeted tyrosine kinase inhibitors (sunitinib and sorafenib) in mRCC. Furthermore, understanding of the role of the mammalian target of rapamycin (mTOR) pathway in RCC has given rise to the clinical use of mTOR inhibitors, temsirolimus and everolimus. Despite the significant improvement in efficacy compared to immunotherapies, the previous gold standard for treatment of mRCC, resistance to these molecular therapies remains a significant problem [14], [15], [16].

Much of the knowledge of this molecular basis of RCC has been uncovered through study of the primary renal tumour, with little analysis of the differences between the primary tumour and the metastases. Recently, Gerlinger *et al* reported that the genetic signature of the primary tumour may not reflect that of the metastasis [17], however there is little study of these differences at the protein level. Lee *et al* have reported increased Bcl-2 expression [18] and Schultz *et al* have shown greater expression of pAKT, pS6, 4EBP1 and cMYC and lower expression of PTEN in the metastatic RCC compared with primary tumours [19]. It is likely that the key to understanding resistance and developing more effective treatments, is an in depth study of the metastatic tumours, as the aim of treatment is to reduce the metastatic tumour burden. Additionally, primary RCC tumour has a predilection to involve the venous system (renal vein, inferior vena cava and right atrium), this venous tumour thrombus (VTT) is potentially a middle ground between the phenotype of the primary and metastatic tumour.

Immunohistochemistry (IHC) is a key tool in molecular pathology used to aid diagnosis and prognosis in RCC. However, IHC is subjective and only semi-quantitative. The development of Automated Quantitative Analysis (AQUA) of immunofluorescence allows accurate and sensitive in situ protein localisation and quantification, which we have refined for use in RCC [11]. Briefly, this involves immunofluorescent staining for the target protein and to create an epithelial tumour mask, allowing differentiation of the tumour from the stroma, and sub-localisation of the cytoplasm and nuclei. There is subsequent digital image capture, and automated analysis of the images to give continuous scores for target expression. This technique allows greater objectivity and accuracy of protein quantification and prognostication offered by Klatte and colleagues. In this study we aim to use AQUA immunoflurescence to assess the differential protein expression of reported prognostic markers in primary tumour, VTT and metastases in RCC and determine their prognostic role in locally advanced and/or metastatic primary renal cell carcinoma.

## Patients and Methods

### Study Population

Patients who had primary renal cell carcinoma and/or renal vein or inferior vena cava thrombus and/or distant RCC metastases at the time of surgery or at a later date were identified from a prospectively compiled database. Formalin fixed paraffin embedded (FFPE) tumour samples were identified from 177 of these patients who underwent radical nephrectomy between 1983 and 2010, in the Department of Urology, Edinburgh. Where possible, written informed consent was gained for use of tissue surplus to diagnostic requirement and linked anonymised patient data. Ethical approval to use these archived tissues was granted by the Lothian Regional Ethics Committee (08/S1101/41 and 10/S1402/33). Ethical approval 08/S1101/41 permits the distribution of FFPE samples and associated linked anonymised data from the Pathology archive for research without consent as they were collected for diagnostic purposes prior to September 2006. The diagnostic pathway for these tissue samples has now been completed and the archived samples are surplus to this process. Additional tissue from after 2006 was collected and released for use under the ethical approval 10/S1402/33, which is Research Tissue Bank ethical approval held by the SAHSC BioResource on behalf of NHS Lothian. This approval applies the principles of ‘position statement on diagnostic archives releasing tissue for research’ joint statement from the Human Tissue Authority (HTA) and the National Research Ethics Service (NES) of July 2009 to the use of these samples. All pathological staging was reported using the TNM 2002 classification [20] **.** If causes of death were not available in clinical notes these were obtained from the General Registry Office of Scotland.

### Tissue Microarray (TMA) Construction

These tissue samples were used to construct a TMA [21]. A minimum of three representative replicate cores, 0.6 mm in diameter, from each patient’s primary tumour, VTT and metastases were taken after review and marking of the hematoxylin and eosin stained slides and blocks by a Consultant Pathologist. Adequate tissue was not available from all primary tumours and metastases. In total, 1980 cores were taken and distributed over 12 slides from the 177 patients representing 163 primary tumours, 103 VTTs and 69 metastases. This included 29 patients with primary tumours only; 79 with matched primary and VTTs; 24 with matched primary tumours, VTTs and metastases; 31 with matched primary tumours and metastases and 14 patients with unmatched metastases only.

### Immunofluorescence

**Figure 1:**
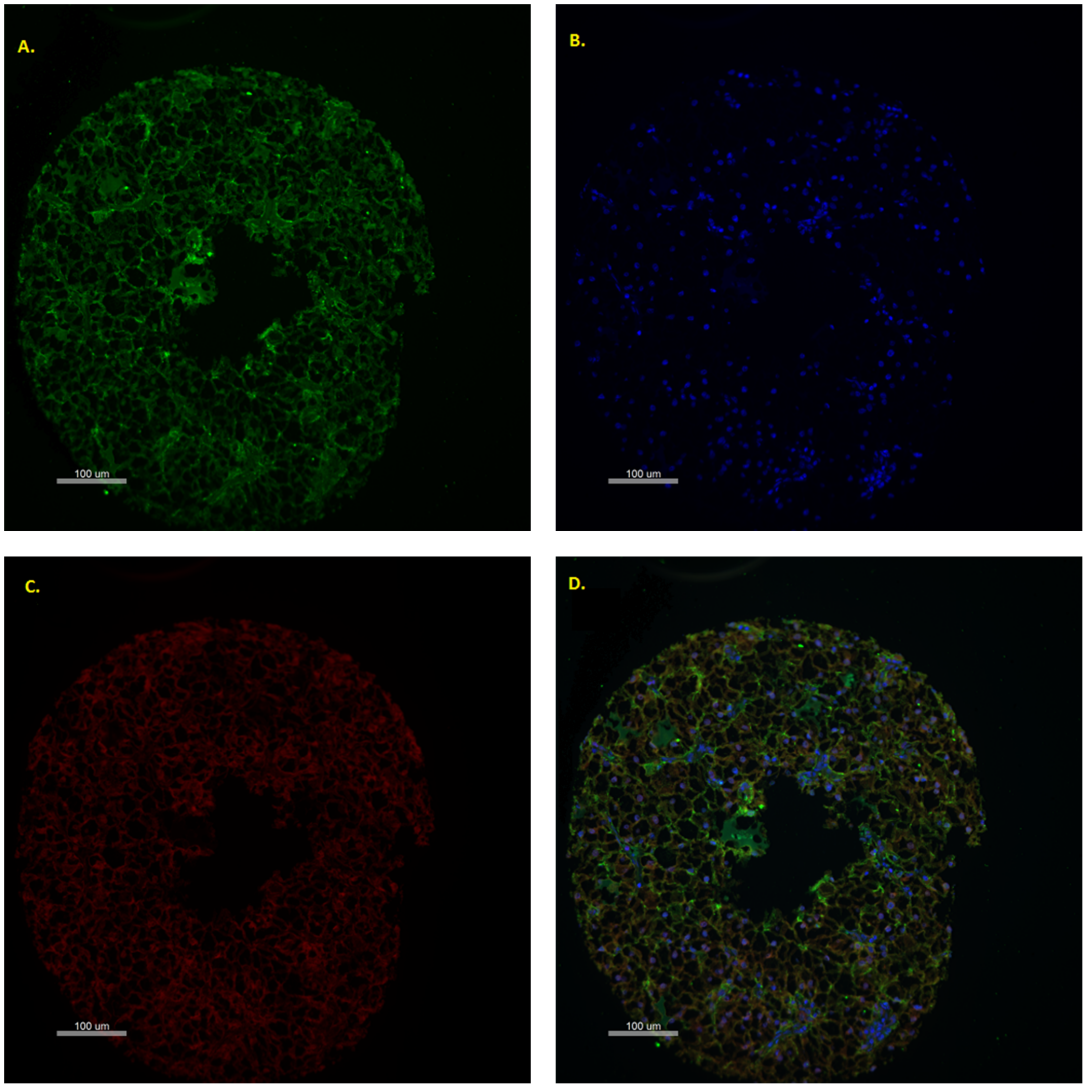
AQUA Images of renal cell carcinoma. Representative TMA core showing green cytoplasmic staining with a combination of cytokeratin and pancadherin (A), blue nuclear staining with DAPI (B), red target staining (C, in this case VEGFR1) and all three compartments combined (D). Quantitative assessment and compartment localisation of target expression is measured through calculating the sum of target pixel intensity divided by the compartment area and normalized for exposure time.

TMA slides were de-waxed in xylene and then rehydrated in sequentially dilute ethanol solutions. Antigen retrieval was conducted by heating the slides in a pressure cooker for 5 minutes in Tris-EDTA pH9.0 or Sodium Citrate pH6.0. Endogenous peroxidase activity was blocked by treating the slides in 3% hydrogen peroxide for 10 minutes and non-specific binding reduced by incubation in serum-free protein block (Dako, X0909) for 10 minutes. Slides were incubated with the primary target antibody, for the protein of interest, for 1 hour at room temperature. Primary antibodies are detailed in table 1. Thereafter the slides were incubated overnight at 4°C with the appropriate antibodies to define the tumour mask. These were pan-cadherin (Cell Signalling, 4068, 1∶100) when using a rabbit primary antibody or a combination of pan-cadherin (Sigma-Aldrich, C1821, 1∶750) and CK5/6/8/18 (Novocastro, 6003168, 1∶100) when using a mouse primary antibody [11]. The slides were then incubated for 1.5 hours with Alexa Fluor 555-conjugated antibody (Invitrogen A21422 and A21428, 1∶25) and horseradish peroxidase–decorated dextran-polymer backbone antibody (EnVision,Dako). Slides were then incubated for 10 minutes with Cy5-Tyramide (HistoRx, AQUAntiplex tube F, 1∶50), which activated the horseradish peroxidase and allowed visualisation of the primary antibody. DAPI (4′, 6-diamidino-2-phenylindole, Invitrogen, P36931) counterstain was used to visualise the nuclei. A representative core is seen in figure 1.

**Table 1:** Primary (target) antibodies used.

Antibody	Catalogue number	Species	Dilution
Ki67	Dako M7240	Mouse	1∶50
P53	Calbiochem OP43	Mouse	1∶200
VEGFR1	Cell Signalling 2893s	Rabbit	1∶100
VEGFD	Abcam ab95442	Rabbit	1∶200
SNAIL	Cell Signalling 3895	Mouse	1∶50
SLUG	Cell Signalling 9885	Rabbit	1∶50

### Automated Quantitative Analysis

Monochromatic images of each TMA core were captured at 20× objective using an Olympus AX-51 epifluorescence microscope (Olympus, Pennsylvania, USA). If the epithelium comprised less than 5% of the total core area, the core was excluded from analysis. High-resolution digital images were analysed by the AQUAnalysis software (HistoRx, Conneticut, USA), to determine target protein expression overall and in the cytoplasmic and nuclear compartments by calculating the sum of target pixel intensity divided by the compartment area and normalized for exposure time.

### Statistical Analysis

Appropriate compartment AQUA scores were normalised by median centring for each TMA to allow inter-TMA comparisons and then the mean tissue score taken from replicate cores. X-tile was used for determining the cut off for defining high and low protein expression in the primary tumour, for Kaplan Meier analysis [22]. Cancer specific survival (CSS) was determined by Kaplan Meier analysis and comparisons made by log-rank test. Predictive significance of protein expression and clinico-pathological factors for CSS was determined using Cox univariate and multivariate regression analysis. Correlation of protein expression with standard pathological factors was assessed with Pearson correlation analysis. Differential protein expression between the primary tumour, renal vein tumour thrombus and metastatic tissue was assessed using the Mann-Whitney test. Statistical analysis was performed using PAWS version 18.0 (SPSS Inc., Chicago, IL, USA), p<0.05 was taken to indicate significance.

## Results

The clinico-pathological features associated with the patients’ primary RCC are shown in Table 2. The median patient age was 64 years and the male to female ratio was 1.5∶1. There was no significant difference in the laterality of tumours and the mean tumour size was 8.7 cm (range: 1.5–16 cm). The majority of tumours (n = 138; 88%) were histologically clear cell, while 14 (9.8%) were papillary and the remainder were other rarer subtypes. Most cases were pathological stage T3b (n = 107, 73.4%). 29 (15.8%) cases were pathologically node positive at time of nephrectomy and 13 (7.1%) patients had confirmed metastases at initial diagnosis.

**Table 2:** Clinico-pathological characteristics of primary renal cell cancers.

Characteristic		N	%
Age	Median years(range)	64 (32–94)	
Gender	Male	96	59.6
	Female	65	40.4
Laterality	Left	60	51.7
	Right	56	48.3
Mean tumour size(cm)		8.7 (1.5–16)	
Histological Subtype	Clear cell	135	88.8
	Papillary	14	9.2
	Other	3	2.0
pT Stage	pT1a	6	4.1
	pT1b	4	2.7
	pT2	13	8.9
	pT3a	11	7.5
	pT3b	107	73.4
	pT3c	1	0.7
	pT4	4	2.7
Fuhrman Grade	1	3	2.8
	2	41	38.3
	3	46	43.0
	4	17	15.9
Node Positive		29	15.8
Metastasis at diagnosis		13	7.1

Ki67 and p53 expression was not identified above background levels in 35.8% (n = 59) and 1.2% (n = 2) of primary RCCs respectively. Otherwise protein expression of the other markers was identified in all tumours. Assessment of the relationship between protein expression in the primary tumour and standard pathological parameters revealed increased SNAIL expression correlated with advanced pathological tumour stage (p = 0.001) and increased maximum tumour diameter (p = 0.048). High VEGFR1 and VEGFD were associated with non-clear cell subtype (p = 0.017 and p = 0.018 respectively).

Furthermore, high VEGFR1 in the complete tumour mask, but not in the localised cytoplasmic compartment, was correlated with node positive disease (p = 0.005). There were no other significant correlations between assessed protein expression and standard clinico-pathological variables.

**Figure 2:**
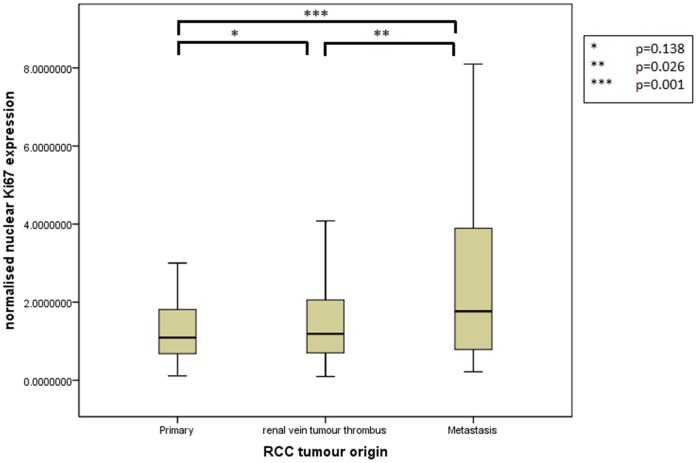
Differential nuclear Ki67 expression between primary, renal vein tumour thrombus and metastatic RCC. Significantly increased expression of nuclear Ki67 shown in the metastases compared to the VTT and primary tumour but no difference in expression between the primary tumour and the VTT.

**Figure 3:**
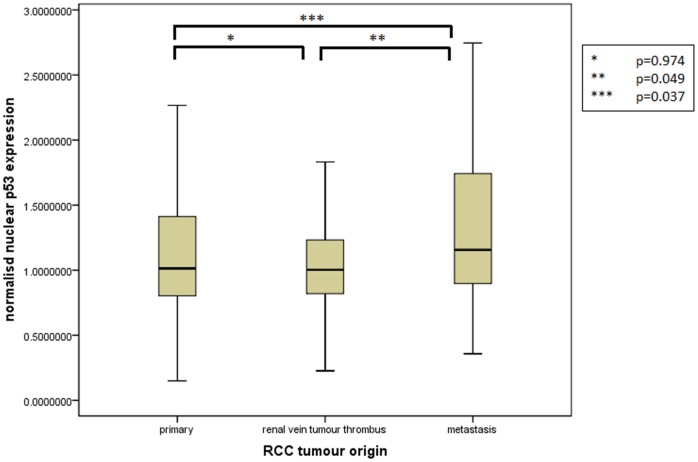
Differential nuclear p53 expression between primary, renal vein tumour thrombus and metastatic RCC. Significantly increased expression of nuclear p53 shown in the metastases compared to the VTT and primary tumour but no difference in expression between the primary tumour and the VTT.

**Figure 4:**
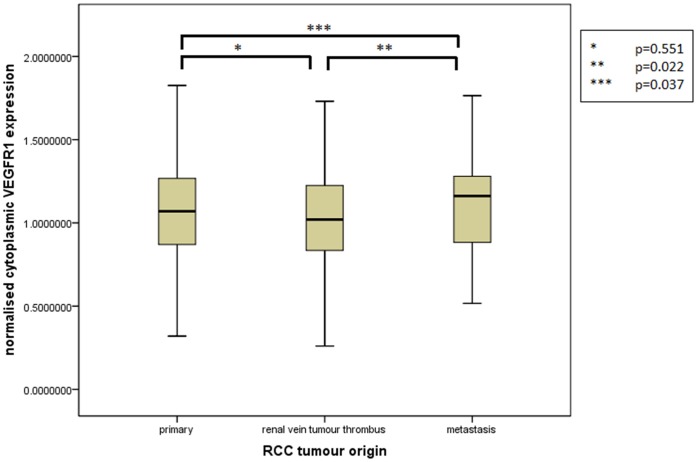
Differential cytoplasmic VEGFR1 expression between primary, renal vein tumour thrombus and metastatic RCC. Significantly increased expression of cytoplasmic VEGFR1 shown in the metastases compared to the VTT and primary tumour but no difference in expression between the primary tumour and the VTT.

**Figure 5:**
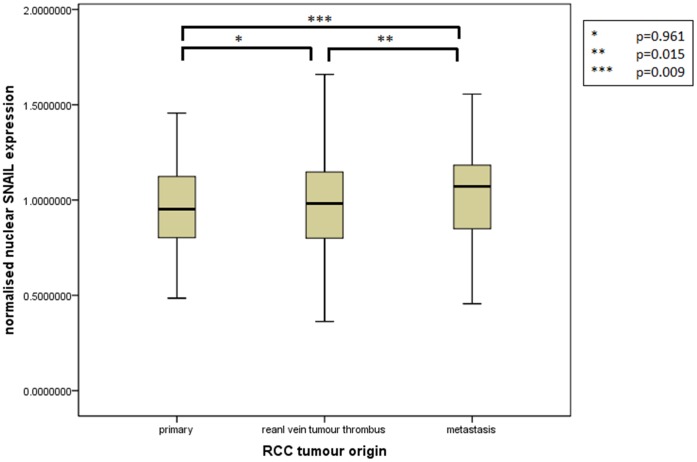
Differential nuclear SNAIL expression between primary, renal vein tumour thrombus and metastatic RCC. Significantly increased expression of nuclear SNAIL shown in the metastases compared to the VTT and primary tumour but no difference in expression between the primary tumour and the VTT.

**Figure 6:**
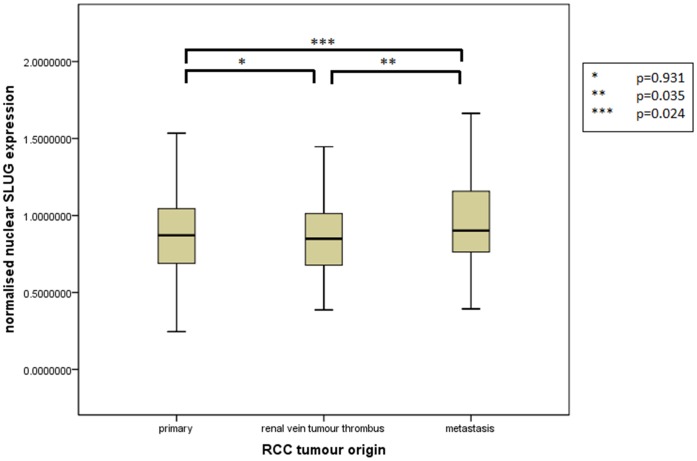
Differential nuclear SLUG expression between primary, renal vein tumour thrombus and metastatic RCC. Significantly increased expression of nuclear SLUG shown in the metastases compared to the VTT and primary tumour but no difference in expression between the primary tumour and the VTT.

**Figure 7:**
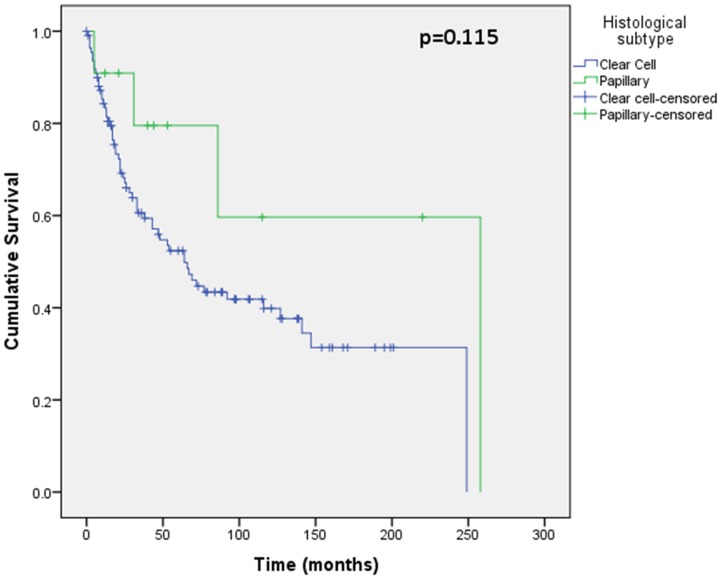
Kaplan Meier curve showing relationship of CSS with histological subtype. No significant difference in CSS is shown between clear cell (n = 111) and papillary RCC (n = 11) tumours.

**Figure 8:**
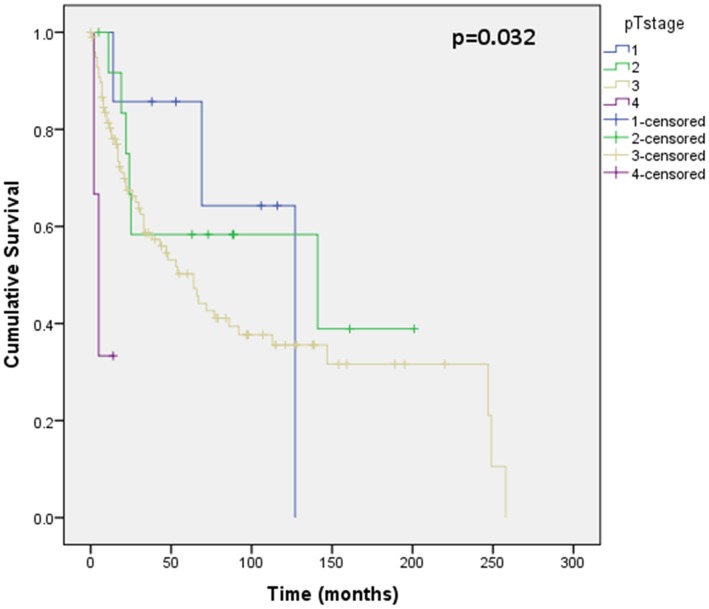
Kaplan Meier curve showing relationship of CSS with pathological tumour stage. CSS was statistically different based on pathological tumour stage on Kaplan Meier analysis. 122 patients were included in this analysis (7 pT stage 1, 13 pT stage 2, 99 pT stage 3 and 3 pT stage 4), others were excluded due incomplete follow-up or pathology data. This shows poorer CSS with worsening pathological tumour stage at diagnosis.

**Figure 9:**
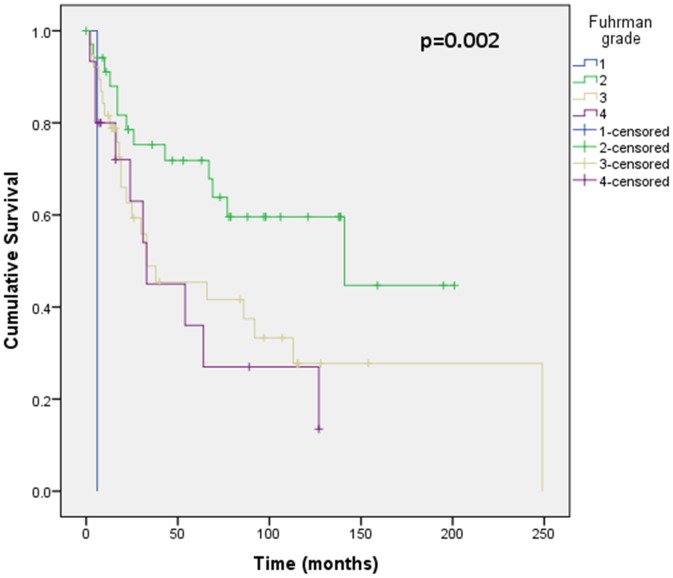
Kaplan Meier curve showing relationship of CSS with Fuhrman grade. This shows statistically different CSS based on Fuhrman grade. 89 patients were included in this analysis (1 grade 1, 35 grade 2, 38 grade 3 and 15 grade 4), others were excluded due incomplete follow-up or pathology data. There is poorest CSS for grade 1, with indistinguishable CSS for grade 3 and 4, and most favourable CSS for grade 2.

**Figure 10:**
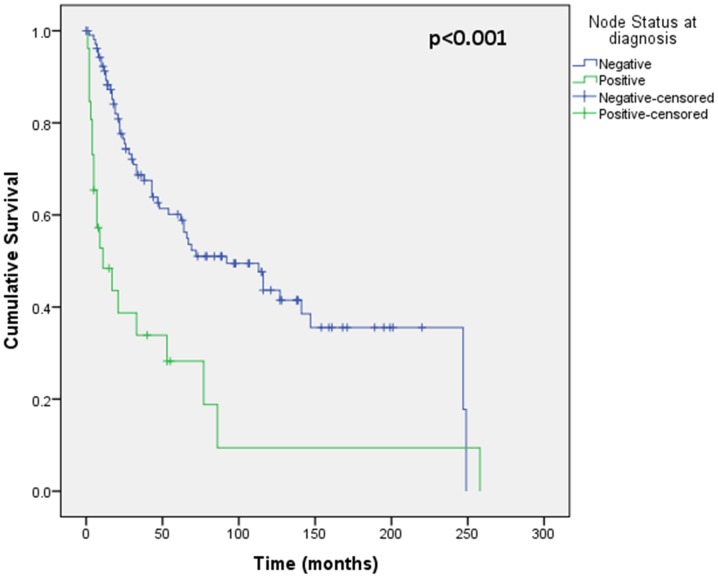
Kaplan Meier curve showing relationship of CSS with node status at diagnosis. Positive node status at diagnosis has significantly worse CSS than node negative disease.

**Figure 11:**
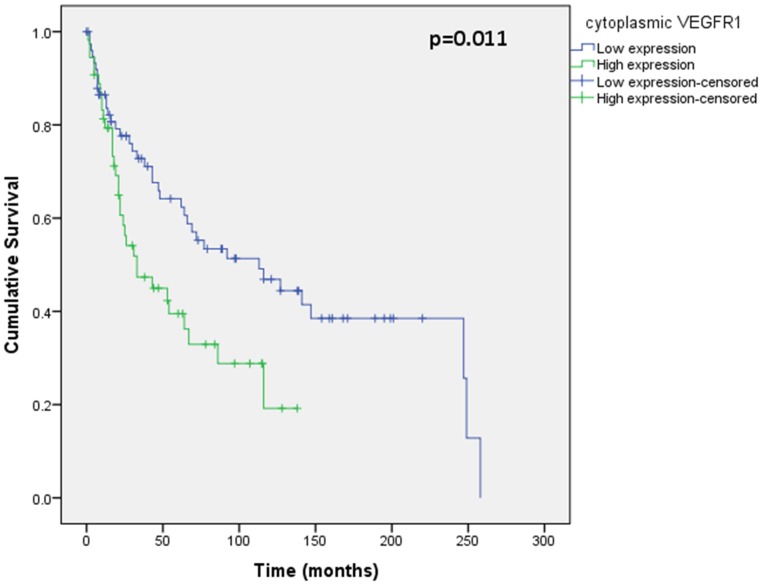
Kaplan Meier curve showing relationship of CSS with cytoplasmic VEGFR1. High VEGFR1 expression in the primary tumour is related to significantly worse CSS than low VEGFR1 expression.

**Figure 12:**
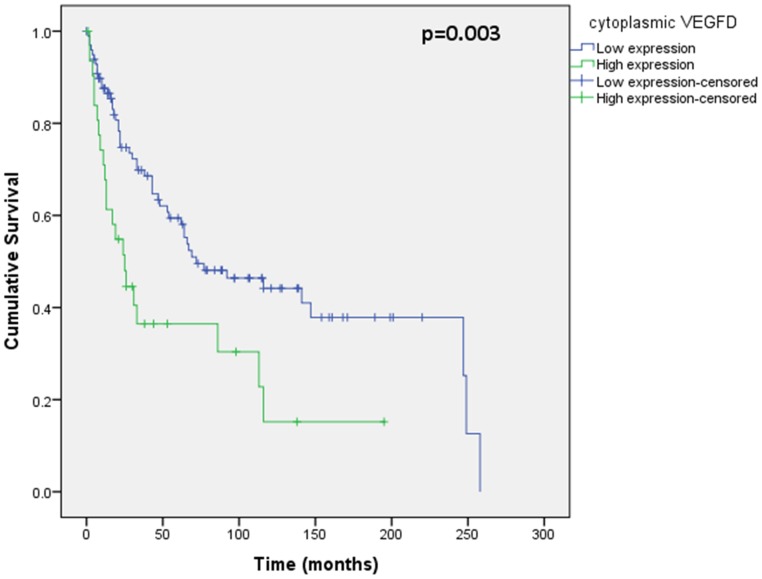
Kaplan Meier curve showing relationship of CSS with cytoplasmic VEGFD. High VEGFD expression in the primary tumour is related to significantly worse CSS than low VEGFD expression.

Assessment of differential protein expression between the primary tumour, VTT and the metastases revealed no significant difference between the primary tumour and VTT but significantly increased expression in the metastases compared to the primary and VTT for Ki67, p53, VEGFR1, SNAIL and SLUG (Figures 2, 3, 4, 5, 6). Accurate survival data was available for 132 patients. Mean follow-up time was 58 months. Histological subgroup did not significantly influence CSS on Kaplan Meier analysis; however, pathological tumour stage (pT stage), Fuhrman grade and node positive disease at diagnosis did predict CSS (figures 7, 8, 9, 10). Survival analysis of the proteomic markers revealed high VEGFR1 and VEGFD to be associated with significantly lower CSS (figures 11 & 12). There was no significant difference in CSS with Ki67, p53, SNAIL and SLUG expression level. Median, predicted 5 and 10 year survival for the pathological variables and proteomic markers are detailed in tables 3 and 4 respectively.

**Table 3:** Histological parameters associated cancer specific survival.

Histological parameter		P-value	Median	5 yr survival	10 yr survival
Histological subtype	Clear cell	0.115	64.0	52.4	39.8
	Papillary		258.0	79.5	59.7
pT stage*	1	0.032	127.0	85.7	64.3
	2		141.0	53.3	58.3
	3		64.0	50.2	35.6
	4		5.0	33.3	–
Fuhrman Grade*	2	0.002	141.0	71.8	59.6
	3		33.0	45.4	27.7
	4		33.0	36.0	–
Node Status*	Negative	<0.001	92.0	60.1	43.7
	Positive		11.0	28.2	9.4

* highlights significant variable.

**Table 4:** Protein related cancer specific survival.

Protein	Expression level	p value	Median	5 yr survival	10 yr survival
Ki67	Low	0.195	72.0	56.3	39.2
	High		25.0	33.6	25.2
p53	Low	0.237	69.0	59.8	39.4
	High		33.0	42.8	32.1
VEGFR1*	Low	0.011	113.0	64.2	46.9
	High		33.0	39.5	19.2
VEGFD*	Low	0.003	72.0	59.4	44.2
	High		25.0	36.5	15.2
SNAIL	Low	0.714	127.0	62.1	51.1
	High		62.0	50.7	33.5
SLUG	Low	0.205	72.0	60.8	35.6
	High		53.0	47.7	36.4

* highlights significant variable.

Those variables which were significant in Kaplan Meier analysis were assessed with Cox multivariate regression analysis, and the clinical variables of Fuhrman grade and node status remained significant, while pT stage, VEGFR1 and VEGFD were not independently prognostic (Table 5).

**Table 5:** Cox multivariate regression analysis of proteomic and clinico-pathological factors in cancer specific survival found to be significant on Kaplan Meier analysis.

Variables	P value	Hazard Ratio	95% Confidence Intervals	
			Lower	Upper
cytoplasmic VEGFR1	0.485	1.3	0.66	2.38
cytoplasmic VEGFD	0.124	1.7	0.87	3.28
Fuhrman Grade*	0.036	1.6	1.03	2.50
pT stage	0.146	1.7	0.83	3.46
Node status at diagnosis*	0.009	2.6	1.27	5.44

* highlights significant variable. Confirming Fuhrman grade and node status to be independently significant on multivariate analysis of predictors of CSS.

## Discussion

This novel study has demonstrated significantly increased expression of Ki67, p53, VEGFR1, SLUG and SNAIL in RCC metastases, compared to the primary tumour and the venous tumour thrombus. Furthermore, primary tumour expression of VEGFR1 and VEGFD can be used to predict significant differences in cancer specific survival in advanced renal cell carcinoma. While VEGFR1 and VEGFD were significant on univariate analysis, superior independent prognostic significance of these markers over standard clinico-pathological parameters in multivariate analysis could not be shown.

To our knowledge, this is the largest reported series of primary and metastatic RCC samples used to construct a TMA for comparative protein analysis, and is the only study to assess the VTT at a molecular level. Additionally, the use of AQUA immunofluorescence allows more accurate and objective protein quantification, an improvement on other published work studying protein expression using the TMA technique. However, we acknowledge this unique and difficult to acquire tissue set still represents a small cohort. This may give rise to type II errors as a result of an underpowered study. In particular this may have prevented significant differences between the primary tumour and VTT protein expression being detected and potential underestimation of the prognostic significance of these markers and known pathological prognostic indicators, especially in multivariate analysis. The markers chosen were based on previously documented prognostic significance, implicating their importance in RCC pathogenesis, however it would be important in the future to perform a large scale unbiased assessment of proteins to assess the scale of difference between primary and metastatic RCC. Nonetheless, significant differences in protein expression of key markers between the primary tumour and metastasis have been shown for the first time.

The difference in Ki67 expression between primary and metastatic lesions has been studied in a number of cancers including breast, colon, gastrointestinal stromal (GIST) and bladder cancer, with contrasting results. In breast and colorectal cancer, higher Ki67 expression in primary tumours compared with nodal and liver metastases respectively, was shown [23], [24], [25], [26]. However, in GIST and bladder tumours, as well as other studies of breast and colorectal carcinoma, the converse or no difference in expression has been demonstrated [27], [28], [29], [30]. The results of the present study have shown a significant increase in Ki67 expression in metastatic RCC tissue compared to primary and VTT RCC, suggesting up regulation of proliferation, which has not been reported previously. Others however have investigated differential p53 expression in primary and metastatic RCC [31], [32], [33]. Due to the extended half-life of mutant p53 compared with wild type p53, mutant rather than wild type p53 is detected with immunohistochemistry [33]. Our work has confirmed the increased expression of mutant p53 in metastatic RCC shown previously, implicating inactivation of p53 in disease progression.

This study has also demonstrated for the first time, increased expression of VEGFR1 in metastatic compared to primary and VTT RCC tissue. VEGF is an important signalling protein involved in angiogenesis and lymphangiogenesis [34], key processes in cancer growth and dissemination; hallmarks of cancer [35]. The up regulation of VEGF through the induction of the hypoxia pathway with mutation of the VHL gene is well accepted in the pathogenesis of ccRCC and has led to the successful development of molecular therapies in the treatment of RCC. However, differential VEGF and VEGFR expression in primary and metastatic RCC has not previously been studied, despite the RCC metastases being the target of VEGF inhibitors. Increased VEGF (rather than VEGFR) mRNA levels were seen in liver metastases compared to primary colorectal tumours [36], while no difference in protein expression of VEGF or VEGFR1 in melanoma and paired metastases has been reported [37]. Our results have confirmed increased expression of VEGFR1 in metastatic RCC, implicating up regulation of angiogenesis and lymphangiogenesis, and supporting the use of VEGF targeted therapies in metastatic disease.

Activating invasion and metastasis is another hallmark of cancer [35] and epithelial to mesenchymal transition (EMT) is thought to be an important process in this, in which a physiological developmental process is reversed. Sarcomatoid de-differentiation is seen in RCC [38], [39], and this may be a phenotypic example of EMT observed by the pathologist. We have shown significantly greater expression of both SLUG and SNAIL proteins in metastatic RCC compared to the primary tumours, as well as a correlation between increased SNAIL expression and increasing tumour size and advancing pT stage. This supports the development of an EMT phenotype at a molecular level in RCC pathogenesis.

This work so far has indicated key proteins may be important in the pathogenesis of RCC and shown significant differences in the protein expression in primary and VTT RCC compared to the metastatic deposits. We hypothesised that the VTT would be an intermediate process between the primary and metastases but were unable to demonstrate this with the proteins assessed. This may be as a result of a number of factors. Firstly, as previously indicated this remains a relatively small cohort, and as such may be underpowered. It is a possibility that the proteins studied are not key to the invasive extension of the primary into a VTT. However, one would intuitively expect markers of proliferation, angiogenesis and EMT to be involved in such a process. Finally, we must accept that a tumour which migrates, adapts and grows in a new environment is different from a tumour which invades locally, and that the VTT may be more representative of an extension of the primary tumour.

As well as differential protein expression, we aimed to assess the prognostic significance of these markers in advanced RCC. We found standard pathological factors remain the most significant variables in predicting cancer specific survival. Nodal disease at presentation has been shown to be prognostic in both otherwise localised disease and metastatic disease independent of other prognostic factors [40], [41], [42]. We have confirmed the significance of this pathological finding in the current study, where positive nodal involvement has the greatest hazard ratio (2.7) on multivariate analysis for CSS.

Fuhrman grade was also found to be significant on univariate and multivariate analysis. Fuhrman grade is based on the assessment of nuclear size, nuclear pleomorphism, and nucleolar prominence [43] and while its prognostic significance in papillary and chromophobe RCC is debated [44] it has been clearly shown to be important in ccRCC [43], [45], [46], [47]. In our analysis, Fuhrman grade 1 had the poorest prognosis and this is likely due the small sample size, while grade 2 disease had significantly prolonged CSS compared with grade 3 and 4, which were indistinguishable. The comparable CSS of patient with Fuhrman grade 3 and 4 disease may be the result of other confounding factors; however, there is currently concern regarding intra- and inter-pathologist reporting variability of Fuhrman grade [44] which has led to the International Society of Urological Pathology (ISUP) recommending replacing Fuhrman grading with ISUP nucleolar grading system, based solely on the nucleolar size as outlined in Fuhrman but without the confounding and confusing issues of nuclear size and shape.

Pathological tumour stage was significant on Kaplan Meier analysis but not multivariate analysis. The small sample size of stages other than pT3, and the high risk nature of the cohort, selected because of renal vein involvement and/or concurrent or subsequent metastases, have prevented identification of the prognostic role of tumour stage clearly identified in localised disease [4], [37], [45], [46]. The small sample size of non-clear cell RCC may also have prevented any significant difference in CSS being identified based on histological subtype (likely type II error), although there was a clear trend of lower CSS with ccRCC.

The prognostic significance of Ki67 and p53 expression has been widely studied in RCC. The proliferation marker, Ki67, has been shown to be associated with higher nuclear grade and worse prognosis in localised ccRCC [12] and mRCC [48]. However, other studies in localised RCC have failed to demonstrate the independent prognostic significance of Ki67, largely because of its close association with tumour grade and stage [49], [50]. Our study of locally advanced and metastatic RCC, although showing a trend of poorer prognosis in higher Ki67 expression, failed to demonstrate any significant difference in CSS based on Ki67 expression. Similarly, the prognostic significance of p53 in RCC is also debated [32], [50], [51], [52], [53], [54], [55], [56], and in our cohort no difference in CSS was seen between high and low expression. Gene expression studies in RCC have shown an EMT signature to be associated with poorer prognosis [57], [58]. Despite these findings, quantitative assessment of SNAIL and SLUG, key proteins in EMT, failed to show any significant difference in CSS.

VEGF expression has also been investigated in RCC by others and found to be prognostic. In particular, VEGFR1 has been found to be associated with worse outcome in localised RCC [12], and our study has confirmed this on univariate analysis in advanced disease. Others have suggested low VEGFD to be associated with worse prognosis [1], [12], however we have seen the converse in advanced disease with poorer outcome associated with higher VEGFD in the primary tumour and we propose this is likely to be the result of up regulation of lymphangiogenesis. However, the loss of significance of these markers in multivariate analysis may be as a result of the very role of VEGF in lymphangiogenesis discussed. This is supported by the correlation between VEGFR1 and node positive disease with the resultant dominant effect of lymph node metastasis in predicting prognosis.

The present study also correlated VEGFR1 and VEGFD with histological subtype, revealing greater expression in pRCC than ccRCC. VEGF expression with histological subtype of RCC is controversial. Ljungberg *et al* studied VEGF and receptor status at the transcriptional level and found higher levels in ccRCC compared with pRCC [59]. It was proposed that this was as a result of the VHL mutation, common in ccRCC but rare in other subgroups, with up regulation of HIF, which has been reported to be greater in ccRCC than other subtypes [60], [61]. However, a proteomic study by Jacobsen *et al* failed to show any difference in VEGF expression between histological subgroups [62], while Dirim et al identified higher VEGF in pRCC compared with ccRCC [63]. There was no significant difference in tumour size, grade or stage between subgroups that may be attributable to the difference found in this study (data not shown). It has been reported that pRCCs are hypovascular, and it is possible that this increase in VEGF is a reflection of the hypoxic microenvironment of the tumour [64]. Our finding further strengthens the importance of VEGF and angiogenesis in progression of RCC and supports the efficacy of angiogenesis inhibitors in all RCC, including non-clear cell subtypes [65].

Although unable to show the independent prognostic significance of selected key proteins above standard pathological parameters in locally advanced RCC, the significance of VEGFR1 and VEGFD on univariate analysis confirms the importance of the hypoxia pathway in RCC pathogenesis. The difference in Ki67, p53, VEGFR1, SLUG and SNAIL between the primary tumour and metastases highlights the importance of proliferation, angiogenesis and EMT in RCC pathogenesis also. We propose greater analysis of the differences between primary tumours and metastases is required to gain a full appreciation of the pathway changes, as these differences may have implications for future work understanding the response to treatment in metastatic disease and overcoming resistance.
